# Scaling clearance in paediatric pharmacokinetics: All models are wrong, which are useful?

**DOI:** 10.1111/bcp.13160

**Published:** 2016-12-02

**Authors:** Eva Germovsek, Charlotte I. S. Barker, Mike Sharland, Joseph F. Standing

**Affiliations:** ^1^ Inflammation, Infection and Rheumatology Section, Great Ormond Street Institute of Child HealthUniversity College London30 Guilford StreetLondonWC1N 1EHUK; ^2^ Paediatric Infectious Diseases Research Group, Institute for Infection and ImmunitySt George's, University of LondonCranmer TerraceLondonSW17 0REUK; ^3^ St George's University Hospitals NHS Foundation TrustBlackshaw RoadLondonUK

**Keywords:** allometric exponent, allometric scaling, children, gentamicin, infants, maturation function, midazolam, neonates, NONMEM, pharmacometrics

## Abstract

**Linked Articles:**

This article is commented on in the editorial by Holford NHG and Anderson BJ. Why standards are useful for predicting doses. Br J Clin Pharmacol 2017; 83: 685–7. doi: 10.1111/bcp.13230

**Aim:**

When different models for weight and age are used in paediatric pharmacokinetic studies it is difficult to compare parameters between studies or perform model‐based meta‐analyses. This study aimed to compare published models with the proposed standard model (allometric weight^0.75^ and sigmoidal maturation function).

**Methods:**

A systematic literature search was undertaken to identify published clearance (CL) reports for gentamicin and midazolam and all published models for scaling clearance in children. Each model was fitted to the CL values for gentamicin and midazolam, and the results compared with the standard model (allometric weight exponent of 0.75, along with a sigmoidal maturation function estimating the time in weeks of postmenstrual age to reach half the mature value and a shape parameter). For comparison, we also looked at allometric size models with no age effect, the influence of estimating the allometric exponent in the standard model and, for gentamicin, using a fixed allometric exponent of 0.632 as per a study on glomerular filtration rate maturation. Akaike information criteria (AIC) and visual predictive checks were used for evaluation.

**Results:**

No model gave an improved AIC in all age groups, but one model for gentamicin and three models for midazolam gave slightly improved global AIC fits albeit using more parameters: AIC drop (number of parameters), –4.1 (5), –9.2 (4), –10.8 (5) and –10.1 (5), respectively. The 95% confidence interval of estimated CL for all top performing models overlapped.

**Conclusion:**

No evidence to reject the standard model was found; given the benefits of standardised parameterisation, its use should therefore be recommended.

## What is Already Known about this Subject

- In children, clearance scales approximately with weight^0.75^ but in neonates and infants, maturation also affects clearance.
- A standardised method for scaling size and postmenstrual age has been proposed but is not always used.
- A systematic comparison of all suggested models is lacking.

## What this Study Adds

- Several published modelling approaches gave similar fits to the same data, but no model out‐performed the standard for all age groups.
- Standardising scaling to a single method does not compromise model fitting and facilitates information sharing.

## Table of Links

**Table:** 

**LIGANDS**
Gentamicin
Midazolam

This Table lists key ligands in this article that are hyperlinked to corresponding entries in http://www.guidetopharmacology.org, the common portal for data from the IUPHAR/BPS Guide to PHARMACOLOGY 1.

## Introduction

**Figure 1:**
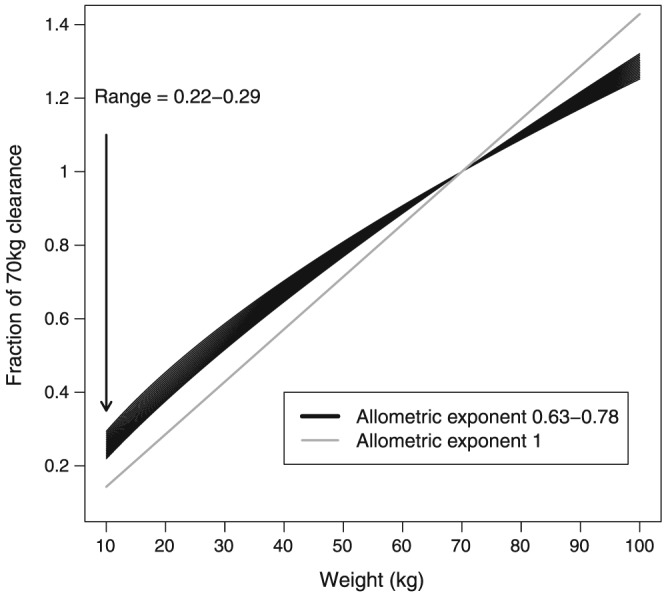
Illustration of the fractional change in clearance compared with using an allometric weight exponent of 0.63–0.78

Smaller people need smaller absolute doses. Since the 1950s paediatricians have recognised that drug clearance (CL), and usually therefore dose requirements (which depend on drug exposure, i.e. area under the curve [AUC]), scales with body surface area rather than body weight 2. Body surface area can be approximated by raising weight to a power of 0.67, and the approach of relating a biological parameter with weight raised to some power is typically known as allometric scaling. The fact that CL scales in this way means that children will have higher dose requirements on a (linear) mg kg^–1^ basis compared to adults (see Figure 1). In 1950, Crawford *et al.* 2, and then almost 5 decades later, Holford 3, highlighted the parallels between weight and CL with the relationship of weight and basal metabolic rate. Basal metabolic rate and how it scales with weight has been studied for over a century and various “correct” values have been derived for the exponent with 0.75 4 and 0.67 5 being the two commonly argued “true” values. A comprehensive review summarising various mathematical descriptions of these observations, along with discussion on whether “basal”, “field”, or any other variety of metabolic rate should be used to infer drug CL scaling was recently provided by Mahmood 6.

Rather than focusing on cross‐species studies of metabolic rate however, when considering drug CL paediatric pharmacologists will be more interested in how eliminating organ function scales with size, and how drug CL scaled with size in previous studies. Rhodin *et al.* 7 found that in children and adults, glomerular filtration scales with weight raised to a power of 0.63, and of course paediatric nephrologists scale their reports of glomerular filtration rate by body surface area 8, 9. Concerning hepatic CL, Johnson *et al.* 10 found liver volume (and therefore hepatic blood flow 11) scales with weight raised to a power of 0.78. We can see from this that biological processes concerned with CL scale with weight raised to a power of 0.63–0.78 in children.

Whilst allometric scaling for size with an exponent of around 0.63–0.78 is widely accepted to be a useful approach to describe or predict CL in children 6, 12, it often does not perform as well in neonates and infants due to the maturation of drug eliminating processes. Two main approaches have been developed over recent years to account for this. The first is to use allometric weight scaling with a single fixed or estimated exponent with CL further multiplied by a maturation factor to adjust for age. This maturation factor is usually a logistic function of age which asymptotes towards 1 with increasing age. The second method is to use/estimate an allometric exponent which changes with either weight or age, for example with a sigmoidal function 13. For both of these approaches a wide variety of maturation functions or functions to vary the allometric exponent have been used.

A popular method for scaling for size and maturation is to fix the allometric weight exponent to 0.75, and to use a sigmoidal maturation function driven by postmenstrual age (PMA) (gestational plus postnatal age) such that CL scales in the following manner:

(1) $$\mathrm{CL}={{\mathrm{CL}}_{T}\cdot \left(\frac{\mathrm{WT}}{70}\right)}^{0.75}\cdot \frac{{\mathrm{PMA}}^{\text{Hill}}}{{\mathrm{PMA}}_{50}^{\text{Hill}}+{\mathrm{PMA}}^{\text{Hill}}},$$

where *CL* is drug CL in an individual, *CL*_*T*_ is the typical CL for a 70 kg adult, *WT* is body weight, *PMA*_*50*_ is the PMA (usually in weeks) for CL to reach 50% mature, and *Hill* is the shape parameter. The rationale behind using PMA rather is that preterm neonates may have lower CL in early life due to immaturity of organ function and drug metabolising enzyme expression. This model has been proposed as a standard method for modelling CL in children 12, and its advantages are threefold: firstly the allometric exponent is fixed to a value within the accepted range of plausible values, thereby adding biological prior knowledge to the model without requiring the addition of a parameter. Secondly, the maturation parameters are easy to communicate: PMA_50_ gives the age at which CL is half‐way to being explained solely by weight, and the Hill coefficient describes the steepness of the maturation curve. Thirdly this model is flexible enough to describe slow and rapid (step‐like) maturation and anything in between.

This study aimed to seek evidence to reject the model presented in Equation (1). Our hypothesis was that no published model gives superior fit to this model across all age groups. We sought to test this by systematically reviewing the literature to identify models for maturation and/or size, and comparing their fit to the CL of two typical drugs, gentamicin (cleared almost exclusively by glomerular filtration) and midazolam (cleared almost exclusively by hepatic metabolism).

## Methods

### Gentamicin and midazolam CL data collection

The Medline database was searched using PubMed in (search last updated March 2016) to identify clinical pharmacokinetic (PK) studies where the CL of intravenously administered gentamicin and midazolam was reported. The keywords for the search strategy were: pharmacokinetics, midazolam, and gentamicin, and the filter “humans” was applied. The reference lists of the identified publications were also manually searched.

For each CL value the corresponding ages and weights were extracted from the reports. Since it is known that CL changes rapidly in the first weeks and months of life 7, 14, we did not include CL estimates where a wide age range of subjects (i.e. age a few days or weeks up to >10 years) were analysed together, with only a mean CL estimate provided for the whole group. Only gentamicin CL values that corresponded to age up to 50 years were kept in the dataset to avoid including adult values affected by declining renal function in older subjects. When only weight or age range was reported, the middle of the range was taken as the mean value of the demographic data. In neonatal studies, if only birth weight was reported, this was assumed as current body weight. A gestational age (GA) of 40 weeks was assigned for children and adults that did not have GA reported. Where only age was reported, typical weight for age was calculated using a published model 15. We did not include studies where a disease was known to affect the CL of midazolam or gentamicin.

### Systematic search for models used to scale CL

A systematic literature review was undertaken (last updated in March 2016) using MEDLINE via PubMed, and, additionally, we emailed the NMUsers discussion group (a global discussion forum for users of NONMEM software) 16, to identify models for size and maturation. Search keywords were: allometry, allometric, scaling, pharmacokinetic, and pharmacokinetics. All models were compared to the proposed model (Equation (1)) with a fixed allometric exponent of 0.75 and a sigmoidal maturation function 12. For comparison, we also tested the parsimonious model of a single weight effect with either estimated allometric exponent or the allometric exponent fixed to 0.75 or 0.67.

### Comparison of models for size and maturation

All models were normalized to 70 kg to facilitate parameter comparison. All parameters that were estimated in the original study were also estimated during the model comparison. We also tested the performance of a simple allometric model with either a single fixed (to 0.75 or 0.67) or estimated exponent. Fitting was performed using NONMEM version 7.3 17. Since CL is usually assumed to follow a log‐normal distribution, an exponential residual error model was used.

The Akaike information criteria (AIC), which was given by –2LL + 2p (where –2LL is –2 times the log likelihood reported as the objective function values in NONMEM and p the number of estimated parameters) was calculated for each model to the overall data and split by age as follows: neonates (0–28 days), infants (1–23.9 months), children (2–11.9 years), adolescents (12–18 years) and adults (>18 years). For each age group, the –2LL value for that age group only was used. The difference in AIC between the tested model and the proposed standard model was calculated, with a better performing model being defined as one in which the AIC was lower than the standard. We defined a better‐performing model as one for which the AIC was lower than the standard model in all age groups. Visual predictive checks were created using R version 3.1.0 18. For the five best models (lowest AIC values) the typical CL and 95% confidence interval were generated by simulation of 1000 parameter combinations using the standard errors from the NONMEM covariance step for a typical neonate, infant, child and adolescent.

## Results

In total, 38 19, 20, 21, 22, 23, 24, 25, 26, 27, 28, 29, 30, 31, 32, 33, 34, 35, 36, 37, 38, 39, 40, 41, 42, 43, 44, 45, 46, 47, 48, 49, 50, 51, 52, 53, 54, 55, 56 and 44 57, 58, 59, 60, 61, 62, 63, 64, 65, 66, 67, 68, 69, 70, 71, 72, 73, 74, 75, 76, 77, 78, 79, 80, 81, 82, 83, 84, 85, 86, 87, 88, 89, 90, 91, 92, 93, 94, 95, 96, 97, 98, 99, 100 publications that included reported CL values were identified for gentamicin and midazolam, respectively. These papers reported a total of 66 and 57 CL values for gentamicin and midazolam, respectively. Four studies including a wide range of neonates, infants and children with only a mean CL estimate provided for the whole group were excluded 101, 102, 103, 104. Similarly, four gentamicin studies including wide adult age ranges (e.g. 16–96 years) were excluded 105, 106, 107, 108. Of the remaining data, a further 10 gentamicin CL values in subjects aged over 50 years were excluded 46, 47, 51, 52, 53, 54, 55. Eight studies 39, 41, 42, 46, 47, 72, 79, 89 did not report subjects' weights, so these were inferred from age as described above. The data used for modelling are presented in Supplementary materials Tables S1 and S2.

The models identified in the literature search that sought to account for changing age and weight relationships in neonates and infants could be split into two main categories: those that, in common with the standard model, add an age function to a fixed or estimated weight function to account for maturation in neonates/infants; and those that use an allometric weight exponent which changes by either age or weight. This change can be fixed predetermined steps or a continuous function. Model structure and estimated parameters are given in Table 1.

**Table 1:** Table of model parameter estimates

No.	Ref	Model equation	Studied population age range	Parameter	Gentamicin	Midazolam
**1**	12	$\mathrm{CL}=\theta_{1}\cdot {\left(\frac{\mathrm{WT}}{70}\right)}^{0.75}\cdot \frac{{\mathrm{PMA}}^{\theta_{2}}}{\theta_{3}^{\theta_{2}}+{\mathrm{PMA}}^{\theta_{2}}}$		θ_1_	5.97 (0.33)	27.4 (0.95)
				θ_2_	4.19 (0.70)	4.04 (0.53)
				θ_3_	45.1 (2.96)	55.4 (4.49)
				RUV	0.075 (0.018)	0.071 (0.012)
**2**		$\mathrm{CL}=\theta_{1}\cdot {\left(\frac{\mathrm{WT}}{70}\right)}^{\theta_{2}}$		θ_1_	9.16 (1.42)	24.9 (1.10)
				θ_2_	1.28 (0.05)	0.82 (0.10)
				RUV	0.13 (0.027)	0.20 (0.056)
**3**		$\mathrm{CL}=\theta_{1}\cdot {\left(\frac{\mathrm{WT}}{70}\right)}^{0.75}$		θ_1_	2.90 (0.31)	24.0 (1.39)
				RUV	0.64 (0.087)	0.19 (0.05)
**4**		$\mathrm{CL}=\theta_{1}\cdot {\left(\frac{\mathrm{WT}}{70}\right)}^{0.667}$		θ_1_	2.55 (0.31)	23.1 (1.35)
				RUV	0.81 (0.122)	0.19 (0.053)
**5**	119	$\mathrm{CL}=\theta_{1}\cdot {\left(\frac{\mathrm{WT}}{70}\right)}^{\theta_{2}}\cdot \left(1+\theta_{3}\cdot \mathrm{PNA}\right)$	Neonates	θ_1_	9.16 (1.42)	24.9 (1.10)
				θ_2_	1.28 (0.05)	0.82 (0.10)
				θ_3_	5.8 × 10^−9^ (1.52 × 10^−9^)	5.8 × 10^−9^(4.13 × 10^−9^)
				RUV	0.13 (0.027)	0.20 (0.056)
**6**	28	$\mathrm{CL}=\theta_{1}\cdot {\left(\frac{\mathrm{WT}}{70}\right)}^{\theta_{2}}+\theta_{3}\cdot \mathrm{PNA}$	Neonates	θ_1_	8.56 (1.29)	24.9 (1.10),
				θ_2_	1.26 (0.04)	0.82 (0.10)
				θ_3_	0.02 (0.064)	6.6 × 10^−8^ (2.47 × 10^−8^)
				RUV	0.13 (0.027)	0.20 (0.056)
**7**	120	$\mathrm{CL}=\theta_{1}\cdot {\left(\frac{\mathrm{WT}}{70}\right)}^{0.75}\cdot \left(1+\theta_{2}\cdot \left(\mathrm{PMA}-40\right)\right)$	Neonates	θ_1_	1.51 (0.17)	24.0 (1.39)
				θ_2_	0.0065 (0.0035)	5.0 × 10^−11^ (1.43 × 10^−10^)
				RUV	0.34 (0.087)	0.20 (0.050)
**8**	121	$\mathrm{CL}=\theta_{1}\cdot {\left(\frac{\mathrm{WT}}{70}\right)}^{0.75}\cdot e^{\theta_{2}\cdot \left(\mathrm{PMA}-40\right)}$	Neonates	θ_1_	2.10 (0.37),	24.0 (1.39)
				θ_2_	0.00077 (0.00048)	5.0 × 10^−11^ (2.79 × 10^−11^)
				RUV	0.55 (0.069)	0.20 (0.050)
**9**		$\mathrm{CL}=\theta_{1}\cdot {\left(\frac{\mathrm{WT}}{70}\right)}^{\theta_{2}}\cdot \frac{{\mathrm{PMA}}^{\theta_{3}}}{\theta_{4}^{\theta_{3}}+{\mathrm{PMA}}^{\theta_{3}}}$		θ_1_	5.86 (0.58)	25.8 (0.98)
				θ_2_	0.72 (0.097)	0.57 (0.053)
				θ_3_	4.25 (0.67)	3.9 (0.46)
				θ_4_	46.1 (4.88)	68.3 (6.58)
				RUV	0.075 (0.018)	0.059 (0.0090)
**9b**	7	$\mathrm{CL}=\theta_{1}\cdot {\left(\frac{\mathrm{WT}}{70}\right)}^{0.632}\cdot \frac{{\mathrm{PMA}}^{3.33}}{55.4^{3.33}+{\mathrm{PMA}}^{3.33}}$	Neonates–adults	θ_1_	5.59 (0.22)	–
				RUV	0.086 (0.031)	
**10**	122	$\mathrm{CL}=\theta_{1}\cdot {\left(\frac{\mathrm{WT}}{70}\right)}^{0.75}\cdot \left(1-\theta_{2}\cdot e^{-\left(\mathrm{PMA}-40\right)\cdot \frac{\ln2}{\theta_{3}}}\right)$	Neonates–adults	θ_1_	5.98 (0.34)	27.0 (0.94)
				θ_2_	0.65 (0.037)	0.71 (0.060)
				θ_3_	27.1 (4.90)	28.9 (8.51)
				RUV	0.08 (0.023)	0.091 (0.019)
**11**	123	$\mathrm{CL}=\theta_{1}\cdot {\left(\frac{\mathrm{WT}}{70}\right)}^{0.75}\cdot \left(\theta_{2}+\left(1-\theta_{2}\right)\cdot \left(1-e^{-\mathrm{PNA}\cdot \theta_{3}}\right)\right)$	Infants	θ_1_	5.77 (0.34)	27.1 (0.95)
				θ_2_	0.21 (0.018)	0.11 (0.020)
				θ_3_	2.39 (1.53)	2.63 (0.74)
				RUV	0.097 (0.017)	0.078 (0.013)
**12**	109	$\mathrm{CL}=\theta_{1}\cdot {\left(\frac{\mathrm{WT}}{70}\right)}^{0.75}\cdot \left(\theta_{4}+\left(1-\theta_{4}\right)\cdot \frac{{\mathrm{PNA}}^{\theta_{2}}}{\theta_{3}^{\theta_{2}}+{\mathrm{PNA}}^{\theta_{2}}}\right)$	Neonates–children	θ_1_	5.91 (0.39)	27.2 (0.96)
				θ_2_	1.10 (0.17)	7.3 (0.19)
				θ_3_	0.29 (0.17)	0.102 (0.0025)
				θ_4_	0.21 (0.019)	0.12 (0.015)
				RUV	0.097 (0.016)	0.070 (0.011)
**13**	6	$\mathrm{CL}=\theta_{1}\cdot {\left(\frac{\mathrm{WT}}{70}\right)}^{b}$ ; *b*: 1.2 ≤ 3 mo; 1.0 > 3 mo–2 y; 0.9 > 2–5 y; 0.75 > 5y	Neonates–children	θ_1_	6.67 (0.25)	27.3 (1.41)
				RUV	0.08 (0.024)	0.15 (0.039)
**14**	6	$\mathrm{CL}=\theta_{1}\cdot {\left(\frac{\mathrm{WT}}{70}\right)}^{b}$; *b*: 1.25 ≤ 9 kg; 0.76 > 9 kg	Neonates–children	θ_1_	7.64 (0.337)	27.8 (1.52)
				RUV	0.11 (0.032)	0.17 (0.059)
**15**	124	$\mathrm{CL}=\theta_{1}\cdot {\left(\frac{\mathrm{WT}}{70}\right)}^{b}$ ; *b*: *θ*_2_ ≤ 16.5 kg; *θ*_3_> 16.5 kg	Neonates–adults	θ_1_	8.54 (2.08)	24.6 (1.05)
				θ_2_	1.26 (0.075)	0.85 (0.12)
				θ_3_	1.12 (0.28)	0.67 (0.070)
				RUV	0.13 (0.027)	0.20 (0.055)
**16**	116	$\mathrm{CL}=\theta_{1}\cdot {\left(\frac{\mathrm{WT}}{70}\right)}^{b}$, *b* = *θ*_2_ ⋅ *WT*^*θ*3^	Neonates–adults	θ_1_	6.08 (0.57)	25.4 (0.985)
				θ_2_	1.25 (0.026)	1.38 (0.033)
				θ_3_	–0.13 (0.024)	–0.27 (0.033)
				RUV	0.08 (0.018)	0.10 (0.022)
**17**	13	$\mathrm{CL}=\theta_{1}\cdot {\left(\frac{\mathrm{WT}}{70}\right)}^{b}$, $b=\theta_{3}-\frac{\theta_{5}\cdot {\mathrm{WT}}^{\theta_{2}}}{{\theta_{4}}^{\theta_{2}}+{\mathrm{WT}}^{\theta_{2}}}$	Neonates–adults	θ_1_	5.31 (0.35)	26.0 (0.98)
				θ_2_	1.09 (0.29)	13.2 (8.22)
				θ_3_	1.23 (0.058)	1.35 (0.023)
				θ_4_	17.8 (3.54)	7.2 (0.39)
				θ_5_	1.26 (0.049)	0.74 (0.044)
				RUV	0.07 (0.022)	0.056 (0.0083)
**18**	125	$\mathrm{CL}=\theta_{1}\cdot {\left(\frac{\mathrm{WT}}{70}\right)}^{b}$, $b=\theta_{3}-\frac{\theta_{5}\cdot {\mathrm{PNA}}^{\theta_{2}}}{{\theta_{4}}^{\theta_{2}}+{\mathrm{PNA}}^{\theta_{2}}}$	Neonates–children	θ_1_	5.64 (0.44)	26.1 (1.00)
				θ_2_	0.55 (0.26)	19.6 (4.81)
				θ_3_	1.21 (0.032)	1.36 (0.025)
				θ_4_	2.10 (7.84)	0.017 (0.00046)
				θ_5_	0.95 (0.90)	0.72 (0.046)
				RUV	0.07 (0.014)	0.056 (0.0080)

CL, clearance; AIC, Akaike information criterion; θ_1_, typical value of clearance for a 70‐kg adult; b allometric exponent; WT, body weight in kg; PNA, postnatal age in years; PMA, postmenstrual age in weeks, mo, months; y, years. All θs represent estimated parameters, results are presented as mean (standard error). RUV is residual unexplained variability.

**Figure 2:**
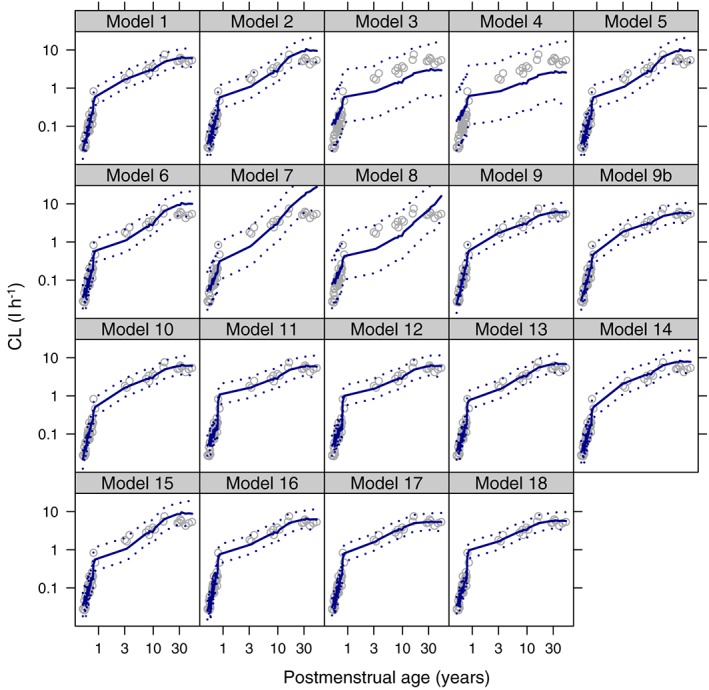
Gentamicin visual predictive checks for each model. Grey open circles are the observed clearance values, the blue solid line is the median simulated model prediction, the dotted blue lines are the 2.5^th^ and 97.5^th^ percentiles of the simulated model predictions. Log–log scale used to aid visualisation of the neonatal period

**Figure 3:**
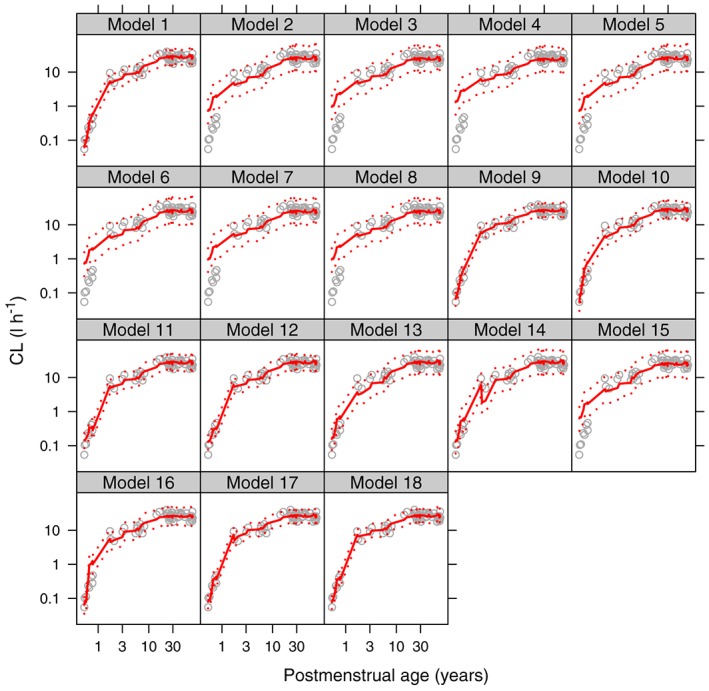
Midazolam visual predictive checks for each model. Grey open circles are the observed clearance values, the red solid line is the median simulated model prediction, the dotted red lines are the 2.5^th^ and 97.5^th^ percentiles of the simulated model predictions. Log–log scale used to aid visualisation of the neonatal period

Change in AIC from the standard model are presented in Table 2, and a visual predictive check of observed CL values with model predictions given in Figures 2 and 3. The model comparisons showed that models with a sigmoidal‐type relationship for neonatal and infant maturation fitted best and that there was very little difference in the fit of these models to the observed CL values (Figures 2 and 3). No model gave consistently better results than model 1 in all age groups based on AIC (Table 2). In Table 3 the CL values and their uncertainty for each age group from the five best models are presented.

**Table 2:** Numerical results showing the change in Akaike information criteria (AIC) between the tested models and the standard model

No.	Gentamicin						Midazolam					
	AIC	AIC Neonates	AIC Infants	AIC Children	AIC Adolescents	AIC Adults	AIC	AIC Neonates	AIC Infants	AIC Children	AIC Adolescents	AIC Adults
**1**	0	0	0	0	0	0	0	0	0	0	0	0
**2**	31.8	6.9	–6.0	10.2	–3.8	16.5	73.8	53.2	–3.7	4.4	–1.5	13.4
**3**	147.1	111.5	–7.3	18.4	3.5	5.0	72.9	57.4	–7.1	0.4	–3.2	9.4
**4**	170.5	131.9	–6.2	17.2	4.4	7.2	76.8	63.4	–7.7	–0.8	–3.0	9.0
**5**	33.8	8.9	–4.0	12.2	–1.8	18.5	75.8	55.2	–1.7	6.4	0.5	15.4
**6**	33.7	8.6	–3.9	11.6	–1.8	19.3	75.8	55.2	–1.7	6.4	0.5	15.4
**7**	97.9	36.0	3.4	18.5	–1.4	33.3	74.9	59.4	–5.1	2.4	–1.2	11.4
**8**	129.9	78.5	–5.3	31.0	0.7	17.0	74.9	59.4	–5.1	2.4	–1.2	11.4
**9**	1.9	2.1	2.3	1.9	2.3	1.3	–9.2	–1.8	0.2	1.3	3.0	–3.9
**9b**	3.8	–4.4	4.4	–3.3	–3.5	–5.4	–	–	–	–	–	–
**10**	4.1	–0.6	4.2	0.4	–0.2	0.4	15.6	9.0	3.3	1.1	–0.1	2.3
**11**	16.7	18.2	–3.4	1.5	0.2	0.2	6.1	7.6	–2.0	0.0	0.1	0.3
**12**	18.7	19.7	–1.5	3.8	1.9	2.7	0.6	5.0	–1.7	1.9	2.3	1.2
**13**	0.8	3.2	–11.4	–1.7	–5.9	0.6	43.3	11.9	7.7	3.1	–4.7	9.3
**14**	18.2	3.1	–3.2	1.8	–6.1	6.7	49.3	5.8	19.9	–1.1	–4.8	13.6
**15**	33.2	9.2	–3.5	13.9	–1.9	15.5	73.7	52.1	–0.1	7.4	0.2	14.1
**16**	6.7	8.3	–6.3	4.3	–0.6	1.0	20.6	23.3	–2.9	1.4	–0.3	–1.0
**17**	0.4	9.4	–1.6	4.5	3.9	0.1	–10.8	1.5	–1.7	1.9	5.3	–1.8
**18**	–4.1	3.8	–0.4	3.4	4.1	1.1	–10.1	0.7	–0.3	1.5	5.4	–1.4

AIC is Akaike information criterion (values are relative to AIC values for Model 1, negative values indicate a better fit than Model 1).

**Table 3:** Parameter estimates (95% confidence interval) for the four models with lowest global AIC for a 1‐day‐old term neonate weighing 3.5 kg, a 1‐year‐old infant weighing 9 kg, a 5‐year‐old child weighing 18 kg and a 12‐year old‐adolescent weighing 39 kg

	Models in order of overall AIC	Typical CL (95%CI) l h^–1^			
		Neonate	Infant	Child	Adolescent
**Gentamicin**	Model 18	0.16^a^	1.03^a^	2.42^a^	4.15^a^
	Model 1	0.24 (0.17,0.32)	1.21 (0.96,1.44)	2.15 (1.76,2.55)	3.85 (3.13 4.59)
	Model 17	0.25 (0.05,1.52)	1.00 (0.30,3.00)	2.36 (1.07,4.83)	4.25 (2.82,6.44)
	Model 13	0.18 (0.16,0.21)	0.86 (0.73,0.98)	1.97 (1.68,2.25)	4.28 (3.65,4.88)
	Model 9	0.23 (0.04,1.54)	1.27 (0.35,4.50)	2.16 (0.93,5.03)	3.84 (2.47,6.00)
	Model 9b	0.34 (0.25,0.45)	1.54 (1.22,1.86)	2.53 (2.06,2.99)	4.12 (3.38,4.92)
**Midazolam**	Model 17	0.46 (0.19,1.10)	6.48 (2.49,16.7)	11.6 (5.56,24.1)	18.3 (13.6,24.4)
	Model 18	0.44^a^	7.02^a^	10.9^a^	18.0^a^
	Model 9	0.51 (0.11,2.12)	6.07 (2.25,16.5)	11.9 (6.56 20.7)	18.7 (14.4,25.0)
	Model 1	0.62 (0.38,0.92)	5.20 (4.49,5.73)	9.88 (9.22,10.6)	17.6 (16.4,19.0)
	Model 12	0.35^a^	5.84^a^	9.82^a^	17.5^a^
	Model 11	0.34 (−0.44,1.12)	5.42 (3.74 5.99)	9.78 (9.00,10.5)	17.5 (16.2,18.7)

a 95% confidence interval cannot be constructed because uncertainty on a parameter raised to some power means possible values are less than zero

## Discussion

We have compared the fit of all the major types of published models for size and age scaling of CL in children to two datasets, and have found that no model gave a superior fit in all age groups to the proposed standard model. Several recent studies have compared the performance of a single or limited range of models for predicting CL in a limited range of drugs 6, 12, 109, 110. None of these studies has systematically compared all published models, so their relative merits are not apparent, although an impressive number of drugs has been used (44 in the case of Holford *et al.* 12). Prediction of paediatric PK, be it with scaled adult models or physiologically based PK, is useful for study design, but ultimately paediatric PK data need to be collected in order to make dosing decisions. For data fitting, models need to be parsimonious (not estimate too many parameters) in order that parameters are estimated with a reasonable degree of precision, yet flexible enough to describe observed trends. Since weight raised to a fixed power of 0.75 with a sigmoidal PMA maturation function has been shown to give good predictions for a large range of compounds 12, we have therefore sought to challenge this model by direct comparison of its ability to fit the same data as all previous published models. No published model was able to out‐perform the standard model for fitting.

Our result has implications for both new drug development and the study of unlicensed and off‐label medicine use, which remains commonplace 111, 112, 113, 114. Patient recruitment to paediatric PK studies remains a challenge in both these settings, and if the same modelling approach was taken for scaling size and age in all studies, this would allow information to be shared across compounds with similar modes of elimination, and facilitate model‐based meta‐analyses. A body of biological prior information on values for PMA_50_ and Hill would be generated which would have a number of uses: (i) allowing the analysts of small datasets to fix size and maturation models based on literature from the same or similar compounds to search for other potential covariates of interest; (ii) giving journal reviewers and regulators the opportunity to compare estimated parameters with those expected from previous studies on similar compounds; (iii) facilitating the inclusion of *in vitro* information on maturation of drug eliminating enzymes 115; and (iv) allowing the combination of studies without requiring the sharing of raw data using model‐based meta‐analysis.

Unsurprisingly, the models that did not account for age or allow the allometric exponent to change with age or weight (Models 2, 3 and 4 in Table 1) performed poorly, confirming the need to account for both. Also, those models with linear or exponential maturation, which tended to have been developed in neonates (Models 5, 6, 7 and 8), did not fit well suggesting the need for the sigmoidal‐type shape that the Hill coefficient gives. Importantly, should the true maturation shape be exponential or linear over the entire human age range, the sigmoidal model has the flexibility to fit these by allowing the Hill coefficient to be 1 and the *PMA*_*50*_ parameter to be very large. Similarly, if maturation is complete in early gestation, the model also has the possibility to account for this with a low estimate of *PMA*_*50*_.

Although no single model gave a reduced AIC in all age groups, Model 18 (and in the case of midazolam only, Models 17 and 9 also) gave slightly better overall fits. Both Models 17 and 18 had five estimated parameters, whereas Model 9 had four estimated parameters, compared with the three estimated parameters of the standard model. The price of this improved fit was an increase in standard errors and indeed Table 3 shows that for Model 18 it was not possible to construct 95% confidence intervals (CIs) since the uncertainty on θ_4_ meant it could take negative values. We did see a trend towards models having superior fit in infants but worse fit in neonates. The main reasons for this are that either models did not account for maturation, or that postnatal age (PNA) rather than PMA was used and hence gestation was not accounted for, worsening the neonatal fit. Since no model had a globally improved AIC in addition to improved AIC in each age group, we found no evidence to reject the standard model.

Whilst the 95% CI for all the CL estimates in Table 3 overlapped each other, and hence they do not significantly differ, dosing recommendations are usually based on the typical model prediction, and so different doses would have been recommended based on these top five models. To take midazolam as an example of where CL may be used to directly infer dosing, Ince *et al.* 116 reported that the lower end of the target concentration for sedation with midazolam was 250 μg l^–1^. Multiplying this by the CL values in Table 3 we have predicted dose ranges of: 24–44, 144–195, 140–165 and 112–120 μg kg^–1^ h^–1^ for the typical neonate, infant, child and adolescent in the example (note that doses are scaled by kg as this is standard practice in paediatric intensive care). Typically for midazolam, neonatal dose rates are titrated to the nearest 25 μg kg^–1^ h^–1^, whereas in older children titrations are in 50 μg kg^–1^ h^–1^. From this it can be seen that all but the neonatal group, the models would all have predicted the same typical dose when scaled to the nearest 50 μg kg^–1^ h^–1^. Even in the neonatal group, if we exclude Models 18 and 12 because 95% CI could not be constructed, and Model 11 since the neonatal CL value could take negative values, we are left with a much tighter range of predicted doses (32–44 μg kg^–1^ h^–1^).

In the neonatal group, the models with lower AIC than Model 1 were Model 9b for gentamicin and Model 9 for midazolam (Table 2). Both these models were variations on Model 1, in that Model 9b used an allometric exponent of 0.632 (tested for gentamicin since this was the estimated exponent for GFR maturation by Rhodin *et al.* 7), and Model 9 estimated the allometric exponent, and so in the age group where there is potential uncertainty in the midazolam recommended dose rate (see above), the standard model fits best. A contributing factor to the standard model performing well in neonates is the use of PMA rather than weight alone, or PNA. The reason to use PMA rather than PNA ought to be apparent, in that by using PNA, a baby born prematurely would be treated in exactly the same way as a term baby despite the fact eliminating organ function and enzyme expression will be less developed. Similarly, allowing the allometric exponent to change with weight gives identical treatment to babies of the same weight regardless of their gestational age. There will almost certainly be additional increases in CL in the first few days of life in addition to those predicted by gestation, and in situations where rich neonatal data with a range of PNA and PMA are available, it may be possible to identify this effect separately 117. Despite the obvious rationale for using of PMA, several published models did not take this approach.

A possible limitation of this work is that despite systematically comparing all models, these were only tested on two datasets, and we also used some model‐based predictions of CL. To address this we would argue that the standard model has already been evaluated on data from 44 drugs 12, and so to discriminate between models required comparison on the same data. Gentamicin and midazolam were chosen as they each accounted for an example renal and hepatic CL respectively, and there were sufficient intravenous data available in the literature to cover the whole age range. Whilst we would have preferred individual noncompartmental AUC_(0–∞)_ estimates to infer CL from, these are simply not available in all age groups, particularly neonates. Hence we did also use model based CL estimates in narrow age and weight ranges, and consider this should not unduly bias our results since all models were tested on the same data. We also did not only include data from healthy subjects, which are anyhow unavailable for paediatric subjects due to ethical reasons. However, we only included data from studies where a disease did not have a known effect on CL (for example, neonates on extracorporeal membrane oxygenation were shown to have similar midazolam CL to nonextracorporeal membrane oxygenation neonates 63), and also some data from critically ill subjects (such as neonates receiving midazolam 62, who were also shown to have similar CL (for an infant of the same weight) to noncritically ill neonates 59).

Whether to estimate an allometric exponent from PK data was recently explored by McLeay *et al.* 118 in an extensive meta‐analysis. They found an average allometric exponent on CL of 56 drugs to be 0.65 (precision of this estimate was not reported but a histogram of the estimated values shows a 95% CI of approximately 0.1–1.2). This highlights the fact that a size‐related allometric exponent can be difficult to identify, and indeed Model 9, which was the standard model with estimated allometric exponent, did not give a superior overall fit. Our results support the argument that fixing the allometric exponent, thereby adding biological prior information on the effect of body size *a priori*, will allow delineation of size from other important covariates without adding an uncertain parameter and thereby potentially destabilising parameter estimation. The importance of minimising the number of estimated parameters is highlighted by Model 18 for which 95% CI of dosing predictions could not be constructed due to the uncertainty in parameter estimates. Interestingly, Model 13, with only one estimated parameter and cut‐off ages to decrease the allometric exponent with increasing age (effectively fixing both the size and maturation parts of the model), performed well for gentamicin, but less well for midazolam, although it did give similar CL values to the standard model in older subjects. From a point of view of model parsimony, this model may be relatively attractive, but the poor fit for midazolam in neonates and infants suggests that fixed cut‐offs in the maturation applied to all drugs may not be appropriate. However, the performance of Model 9b for gentamicin, which used fixed allometric and maturation parameters from a previous study on GFR maturation 7, shows that using biological prior information based on the mechanism of CL may be a useful approach.

In conclusion, a systematic comparison was undertaken of all published models for scaling CL in children, which were tested against the proposed standard model using a fixed allometric weight exponent of 0.75 and an estimated sigmoidal maturation function based on PMA with parameters of 50% mature value and Hill coefficient. We found no evidence to suggest any significant improvement in model fit can be achieved over use of this standard parametrisation. For the two model drugs, midazolam and gentamicin, maturation clearly followed a sigmoidal‐type pattern, so linear or exponential age‐functions should not be used. Standardising model parameterisation to this single approach will benefit the paediatric PK community by facilitating parameter value interpretation and model sharing across studies of the same drug and between compounds.

## Competing Interests

All authors have completed the Unified Competing Interest form at www.icmje.org/coi_disclosure.pdf (available on request from the corresponding author) and declare: The authors report no financial conflict of interest and this manuscript details work that was not specifically funded, but arose from the PhD projects of E.G. and C.B. supervised by M.S. and J.F.S. E.G. has been supported by an IMPACT PhD studentship from University College London (UCL), and has received funding from the NeoMero study, part of the European Union Seventh Framework Programme for research, technological development and demonstration (Grant Agreement number 242 146), and also from Action Medical Research (grant code SP4650, GN1834). C.I.S.B. is funded as a Clinical Research Fellow by the Global Research in Paediatrics Network of Excellence (GRiP), part of the European Union's Seventh Framework Programme for research, technological development and demonstration (FP7/2007–2013, Grant Agreement number 261 060). M.S. chairs the UK Department of Health expert Advisory Committee on Antimicrobial Resistance and Healthcare Associated Infection, is an independent scientific advisor to NICE (The National Institute for Health and Care Excellence), and also receives institutional academic research grants from the NIHR (National Institute for Health Research) and the European Union. J.F.S. has received funding from United Kingdom Medical Research Council Fellowships (grants G1002305 and M008665), and been supported by the National Institute for Health Research Biomedical Research Centre at Great Ormond Street Hospital for Children NHS Foundation Trust and University College London.

## Supporting information


**Table S1** Gentamicin clearance and covariate values used for modelling
**Table S2** Midazolam clearance and covariate values used for modelling

Supporting info item
