# Supplementary material for: Scaling clearance in paediatric pharmacokinetics: All models are wrong, which are useful?
Source: Br J Clin Pharmacol. 2016 Dec 2;83(4):777–90. doi: 10.1111/bcp.13160 (PMC5346879; doi:10.1111/bcp.13160)
Supplement: Supplementary file 1 — Supporting info item [file BCP-83-777-s001.docx]

**Supplementary Material: A comparison of models for scaling clearance in children**

Tables of included data. References are provided in this document for convenience, but please note this means the reference numbers here differ from the main manuscript.

Table S1: Gentamicin clearance and covariate values used for modelling

| **Reference** | **N** | **GA (weeks)** | **PNA range** | **PNA** | **PMA (weeks)** | **WT (kg)** | **CL (L/h)** |
| --- | --- | --- | --- | --- | --- | --- | --- |
| **Neonates** |  |  | **(days or o.w.)** | **(days)** |  |  |  |
| Knight 2003 [[1](#_ENREF_1)] | 26 | 26.6 | 2 | 2 | 26.9 | 0.94 | 0.03 |
| Knight 2003 [[1](#_ENREF_1)] | 5 | 26.8 | 3 | 3 | 27.2 | 0.9 | 0.03 |
| Dodge 1991 [[2](#_ENREF_2)] | 71 | 28.6 | 4 h (<7 days) | 1 | 28.7 | 1.12 | 0.05 |
| Nielsen 2009 [[3](#_ENREF_3)] | 61 | 28.9 | 1 (0-45) | 1 | 29.0 | 1.4 | 0.03 |
| Low 2015 [[4](#_ENREF_4)] | 3 | 27.7 | 0-28 | 14 | 29.7 | 1.07 | 0.03 |
| Nakae 1988 [[5](#_ENREF_5)] | 19 | 29.6 | 1 | 1 | 29.7 | 1.29 | 0.06 |
| Lulic-Botica 2014 [[6](#_ENREF_6)] | 53 | 27 | 22 (IQR: 13-42) | 22 | 30.1 | 1.28 | 0.06 |
| Nakae 1988 [[5](#_ENREF_5)] | 20 | 29.6 | 4 | 4 | 30.2 | 1.29 | 0.04 |
| Lulic-Botica 2014 [[6](#_ENREF_6)] | 29 | 30 | 4 (IQR: 3.5-4) | 4 | 30.6 | 0.86 | 0.03 |
| Lulic-Botica 2014 [[6](#_ENREF_6)] | 135 | 32 | 4 (IQR: 3-4) | 4 | 32.6 | 1.78 | 0.07 |
| DiCenzo 2003 [[7](#_ENREF_7)] | 139 | 32 | 0-10 | 5 | 32.7 | 1.92 | 0.07 |
| Vervelde 1999 [[8](#_ENREF_8)] | 29 | 32 | 6 (1-7) | 6 | 32.9 | 1.8 | 0.09 |
| Garcia 2006 [[9](#_ENREF_9)] | 200 | 32.2 | 5.49 +/- 5.41 | 5.5 | 33.0 | 1.68 | 0.07 |
| Nakae 1988 [[5](#_ENREF_5)] | 18 | 33 | 1 | 1 | 33.1 | 1.83 | 0.11 |
| Knight 2003 [[1](#_ENREF_1)] | 43 | 32.7 | 3-4 | 3.5 | 33.2 | 2.13 | 0.09 |
| Dodge 1991 [[2](#_ENREF_2)] | 58 | 33.1 | 3.5 h (<7 days) | 1 | 33.2 | 1.9 | 0.09 |
| Knight 2003 [[1](#_ENREF_1)] | 99 | 33.0 | 2 | 2 | 33.3 | 2.03 | 0.08 |
| Nakae 1988 [[5](#_ENREF_5)] | 21 | 33 | 4 | 4 | 33.6 | 1.83 | 0.08 |
| Lanao 2004 [[10](#_ENREF_10)] | 97 | 33.2 | 4.61 (1-26) | 4.6 | 33.9 | 1.93 | 0.13 |
| Fuchs 2014 [[11](#_ENREF_11)] | 1449 | 34 | 1 (0-94) | 1 | 34.1 | 2.17 | 0.09 |
| Lulic-Botica 2014 [[6](#_ENREF_6)] | 19 | 28 | 44 (IQR: 21-75) | 44 | 34.3 | 1.42 | 0.08 |
| Low 2015 [[4](#_ENREF_4)] | 27 | 32.5 | 0-28 | 14 | 34.5 | 1.91 | 0.10 |
| Vervelde 1999 [[8](#_ENREF_8)] | 5 | 33 | 15 (12-24) | 15 | 35.1 | 1.95 | 0.20 |
| Haughey 1980 [[12](#_ENREF_12)] | 11 | 35 | 0.2-3 | 1.6 | 35.2 | 1.82 | 0.11 |
| Botha 2003 [[13](#_ENREF_13)] | 79 | 35.1 | 4.2 (3-7) | 4.2 | 35.7 | 2.06 | 0.07 |
| Lingvall 2005 [[14](#_ENREF_14)] | 277 | 36 | 0 (0-27) | 0 | 36.0 | 2.52 | 0.12 |
| Murphy 1998 [[15](#_ENREF_15)] | 195 | 36 | 0.7 | 0.7 | 36.1 | 2.55 | 0.12 |
| Thomson 1988 [[16](#_ENREF_16)] | 113 | 33.5 | 1-46 | 23.5 | 36.9 | 2.2 | 0.10 |
| Low 2015 [[4](#_ENREF_4)] | 13 | 35.6 | 0-28 | 14 | 37.6 | 2.59 | 0.15 |
| Bhatt-Mehta 2003 [[17](#_ENREF_17)] | 18 | 38 | 0 (few hours) | 0 | 38.0 | 3.3 | 0.17 |
| Izquierdo 1992 [[18](#_ENREF_18)] | 97 | 38.1 | 7.78 (2-30) | 7.8 | 39.2 | 2.95 | 0.20 |
| Mark 2013 [[19](#_ENREF_19)] | 7 | 39.3 | <6h at inclusion | 1 | 39.4 | 3.27 | 0.16 |
| Knight 2003 [[1](#_ENREF_1)] | 64 | 39.3 | 2 | 2 | 39.6 | 3.38 | 0.20 |
| Knight 2003 [[1](#_ENREF_1)] | 30 | 39.2 | 3-4 | 3.5 | 39.7 | 3.14 | 0.16 |
| Low 2015 [[4](#_ENREF_4)] | 70 | 37.7 | 0-28 | 14 | 39.7 | 3.31 | 0.26 |
| Arenas-Lopez 2010 [[20](#_ENREF_20)] | 14 | 40 | 1.8 (0.9-15.2) | 1.8 | 40.3 | 3.3 | 0.11 |
| Cohen 1990 [[21](#_ENREF_21)] | 12 | 40 | 9 (5-16) | 9 | 41.3 | 3.51 ^a^ | 0.20 |
| **Children** |  |  | **(years or o.w.)** | **(years)** |  |  |  |
| Medellin-Garibay 2015 [[22](#_ENREF_22)] | 208 | 40 | 5.8 (1-24) months | 0.48 | 42.1 | 6.4 | 0.83 |
| Arenas-Lopez 2010 [[20](#_ENREF_20)] | 36 | 40 | 14 (5-50) months | 1.17 | 45.1 | 7.9 | 0.47 |
| Bass 1998 [[23](#_ENREF_23)] | 31 ^b^ | 40 | 0.5-4 | 2.25 | 157 | 13.1 ^a^ | 1.81 |
| Shankar 1999 [[24](#_ENREF_24)] | 36 | 40 | 2.5 +/- 0.8 | 2.5 | 170 | 13.6 ^a^ | 1.65 |
| Ho 1995 [[25](#_ENREF_25)] | 13 | 40 | 2.9 (1-5) | 2.9 | 190.8 | 14.9 | 2.44 |
| Postovsky 1997 [[26](#_ENREF_26)] | 30 | 40 | 6.6 +/- 4.1 | 6.6 | 383.2 | 24.1 | 2.82 |
| Bass 1998 [[23](#_ENREF_23)] | 31 ^b^ | 40 | 5-10 | 7.5 | 430 | 25.4 ^a^ | 3.38 |
| Postovsky 1997 [[26](#_ENREF_26)] | 22 | 40 | 7.7 +/- 4.4 | 7.7 | 440.4 | 28.2 | 2.41 |
| Ho 1995 [[25](#_ENREF_25)] | 5 | 40 | 9.0 (6-12) | 9 | 508 | 27 | 3.78 |
| Shankar 1999 [[24](#_ENREF_24)] | 37 | 40 | 9.9 +/- 3.8 | 9.9 | 554.8 | 32.3 ^a^ | 3.39 |
| Bass 1998 [[23](#_ENREF_23)] | 31 ^b^ | 40 | 11-18 | 14.5 | 794 | 51.5 ^a^ | 5.51 |
| Ho 1995 [[25](#_ENREF_25)] | 7 | 40 | 15.3 (13-18) | 15.3 | 835.6 | 53.5 | 7.54 |
| **Adults** |  |  | **(years)** | **(years)** |  |  |  |
| Walker 1979 [[27](#_ENREF_27)] | 10 | 40 | 22-32 | 27 | 1444 | 70.5 | 5.08 |
| el-Sayed 1989 [[28](#_ENREF_28)] | 46 | 40 | 18-40 | 29 | 1548 | 70.6 ^a^ | 5.17 |
| Zaske 1982 [[29](#_ENREF_29)] | 469 | 40 | 21-40 | 30.5 | 1626 | 70.9 ^a^ | 6.24 |
| Simon 1973 [[30](#_ENREF_30)] | 7 | 40 | 21-43 | 32 | 1704 | 77.5 | 5.75 |
| Demczar 1997 [[31](#_ENREF_31)] | 11 | 40 | 38 (18-55) | 38 | 2016 | 73 | 4.21 |
| Gilman 1993 [[32](#_ENREF_32)] | 11 | 40 | 44 +/- 14.1 | 44 | 2328 | 73.4 | 4.98 |
| Zaske 1982 [[29](#_ENREF_29)] | 225 | 40 | 41-60 | 50.5 | 2666 | 72.2 ^a^ | 5.42 |
| el-Sayed 1989 [[28](#_ENREF_28)] ^c^ | 53 | 40 | 41-64 | 52.2 | 2754.4 | 72.2 ^a^ | 3.75 |
| Bianco 1989 [[33](#_ENREF_33)] ^c^ | 40 | 40 | 57.7 +/- 13.6 | 57.7 | 3040.4 | 67.5 | 4.69 |
| Bertino 1991 [[34](#_ENREF_34)] ^c^ | 645 | 40 | 60.3 +/- 18.2 | 60.3 | 3175.6 | 72.9 | 4.61 |
| Lewis 1999 [[35](#_ENREF_35)] ^c^ | 9 | 40 | 64 (53.6-74.3) | 64 | 3368 | 76.7 | 3.40 |
| Goncalves-Pereira 2010 [[36](#_ENREF_36)] ^c^ | 32 | 40 | 68 (IQR: 48-79) | 68 | 3576 | 73 | 3.42 |
| Zaske 1982 [[29](#_ENREF_29)] ^c^ | 245 | 40 | 61-80 | 70.5 | 3706 | 72.4 ^a^ | 4.05 |
| Hilmer 2010 [[37](#_ENREF_37)] ^c^ | 17 | 40 | 74.2 +/- 6.5 | 74.2 | 3898.4 | 76.8 | 3.49 |
| el-Sayed 1989 [[28](#_ENREF_28)] ^c^ | 26 | 40 | 65-92 | 78.5 | 4122 | 72.4 ^a^ | 2.77 |
| Johnston 2014 [[38](#_ENREF_38)] ^c^ | 38 | 40 | 80 (65-96) | 80 | 4200 | 76 | 2.96 |
| Zaske 1982 [[29](#_ENREF_29)] ^c^ | 96 | 40 | 81-97 | 89 | 4668 | 72.5 ^a^ | 3.84 |

^a^ weight was estimated using the formula from [[39](#_ENREF_39)]; ^b^ this is the total number of patients in the study (the number of patients in each age sub-group was not given); ^c^ these clearance reports were not included in the analysis; GA is gestational age, PNA is postnatal age, PMA is postmenstrual age, WT is weight, CL is clearance, range in (), +/- standard deviation, IQR is the interquartile range, o.w. is otherwise.

Table S2: Midazolam clearance and covariate values used for modelling

| **Reference** | **N** | **GA (weeks)** | **PNA range** | **PNA** | **PMA (weeks)** | **WT (kg)** | **CL (L/h)** |
| --- | --- | --- | --- | --- | --- | --- | --- |
| **Neonates** |  |  | **(days)** | **(days)** |  |  |  |
| Lee 1999 [[40](#_ENREF_40)] | 60 | 27 | 4.5 (2-15) | 4.5 | 27.6 | 0.96 | 0.05 |
| Harte 1997 [[41](#_ENREF_41)] | 10 | 27.9 | 2-4 | 3 | 28.3 | 1.05 | 0.10 |
| de Wildt 2001 [[42](#_ENREF_42)] | 24 | 29 | 5.5 (3-11) | 5.5 | 29.8 | 1.02 | 0.11 |
| Jacqz-Aigrain 1992 [[43](#_ENREF_43)] | 15 | 32.8 | 1-5 | 3 | 33.2 | 1.9 | 0.23 |
| Burtin 1994 [[44](#_ENREF_44)] | 187 | 34 | 0-10 | 5 | 34.7 | 2.95 | 0.21 |
| Jacqz-Aigrain 1990 [[45](#_ENREF_45)] | 10 | 37.5 | 2-5 | 3.5 | 38.0 | 3.1 | 0.41 |
| Mulla 2003 [[46](#_ENREF_46)] | 19 | 39.5 | 3.8 (0.5-18) | 3.8 | 40.0 | 3.4 | 0.28 |
| Ahsman 2010 [[47](#_ENREF_47)] | 20 | 40.4 | 0.79 (0.17-5.8) | 0.8 | 40.5 | 3 | 0.47 |
| **Children** |  |  | **(years or o.w.)** | **(years)** |  |  |  |
| Peeters 2006 [[48](#_ENREF_48)] | 24 | 40 | 11.1 (3.2-24.7) months | 0.93 | 88.4 | 9.4 | 9.42 |
| Reed 2001 [[49](#_ENREF_49)] | 5 | 40 | 1 (0.5-2) | 1 | 92 | 8 | 5.44 |
| Mathews 1988 [[50](#_ENREF_50)] | 4 | 40 | 1.3 +/- 0.42 | 1.3 | 107.6 | 8.8 | 4.79 |
| Muchohi 2008 [[51](#_ENREF_51)] | 12 | 40 | 27 (7-39) months | 2.25 | 157 | 10.4 | 8.99 |
| Rey 1991 [[52](#_ENREF_52)] | 6 | 40 | 2.5 (1.74-4) | 2.5 | 170 | 15.2 | 12.0 |
| Mathews 1988 [[50](#_ENREF_50)] | 6 | 40 | 4.7 +/- 2.60 | 4.7 | 284.4 | 15.9 | 8.14 |
| Reed 2001 [[49](#_ENREF_49)] | 13 | 40 | 5.1 (2-12) | 5.1 | 305.2 | 18.9 | 11.3 |
| Mathews 1988 [[50](#_ENREF_50)] | 6 | 40 | 5.2 +/- 2.53 | 5.2 | 310.4 | 18.4 | 13.2 |
| Payne 1989 [[53](#_ENREF_53)] | 56 | 40 | 5.52 (3-10) | 5.52 | 327.0 | 17.3 | 9.46 |
| Salonen 1987 [[54](#_ENREF_54)] | 6 | 40 | 6.07 +/- 4.59 | 6.07 | 355.6 | 21.6 | 11.2 |
| Salonen 1987 [[54](#_ENREF_54)] | 6 | 40 | 6.11 +/- 1.67 | 6.11 | 357.7 | 22.3 | 15.0 |
| Salonen 1987 [[54](#_ENREF_54)] | 6 | 40 | 6.72 +/- 5.94 | 6.72 | 389.4 | 28 | 8.11 |
| Salonen 1987 [[54](#_ENREF_54)] | 3 | 40 | 7.33 +/- 6.66 | 7.33 | 421.2 | 32.8 | 13.1 |
| Tolia 1991 [[55](#_ENREF_55)] | 13 | 40 | 13.5 (6-18) | 13.5 | 742 | 47.3^a^ | 28.4 |
| Reed 2001 [[49](#_ENREF_49)] | 2 | 40 | 15.4 (12-16) | 15.4 | 840.8 | 62 | 34.7 |
| **Adults** |  |  | **(years)** | **(years)** |  |  |  |
| Mandema 1992 [[56](#_ENREF_56)] | 8 | 40 | 22 +/- 1 | 22 | 1184 | 69 | 31.4 |
| Olkkola 1996 [[57](#_ENREF_57)] | 12 | 40 | 19-25 | 22 | 1184 | 76 | 39.7 |
| Wermeling 2006 [[58](#_ENREF_58)] | 12 | 40 | 22.3 (20-29) | 22.3 | 1199.6 | 71.1 | 26.9 |
| Shord 2010 [[59](#_ENREF_59)] | 10 | 40 | 21-26 | 23.5 | 1262 | 70.4 | 31.0 |
| Breimer 1990 [[60](#_ENREF_60)] | 8 | 40 | 24 +/- 2 | 24 | 1288 | 75 | 23.5 |
| Heizmann 1983 [[61](#_ENREF_61)] | 6 | 40 | 22-27 | 24.5 | 1314 | 66 | 19.4 |
| Wong 2010 [[62](#_ENREF_62)] | 16 | 40 | 25.2 (18-42) | 25.2 | 1350.4 | 69.2^a^ | 22.5 |
| Schwagmeier 1998 [[63](#_ENREF_63)] | 8 | 40 | 25.9 (22-30) | 25.9 | 1386.8 | 68.3 | 21.8 |
| Albrecht 1999 [[64](#_ENREF_64)] | 9 | 40 | 24-28 | 26 | 1392 | 77.5 | 23.9 |
| Clausen 1988 [[65](#_ENREF_65)] | 8 | 40 | 27 (23-32) | 27 | 1444 | 68 | 25.6 |
| van Gerven 1997 [[66](#_ENREF_66)] | 10 | 40 | 22-32 | 27 | 1444 | 78 | 28.2 |
| Greenblatt 1984 [[67](#_ENREF_67)] | 10 | 40 | 27.9 +/- 1.0 | 27.9 | 1490.8 | 68.8 | 32.0 |
| Balson 1996 [[68](#_ENREF_68)] | 8 | 40 | 28 +/- 8 | 28 | 1496 | 72.1 | 21.3 |
| Greenblatt 1984 [[67](#_ENREF_67)] | 10 | 40 | 28.5 +/- 1.3 | 28.5 | 1522 | 58.5 | 33.1 |
| Hostler 2010 [[69](#_ENREF_69)] | 6 | 40 | 28.5 (19-39) | 28.5 | 1522 | 78.8 | 21.2 |
| Platten 1998 [[70](#_ENREF_70)] | 12 | 40 | 30 +/- 8 | 30 | 1600 | 72 | 27.1 |
| Bancke 2015 [[71](#_ENREF_71)] | 25 | 40 | 30 (18-42) | 30 | 1600 | 77.1 | 17.6 |
| Garg 2012 [[72](#_ENREF_72)] | 24 | 40 | 37 (18-60) | 37 | 1964 | 71.7 ^a^ | 24.0 |
| Greenblatt 1984 [[67](#_ENREF_67)] | 20 | 40 | 37.5 +/- 3.3 | 37.5 | 1990 | 65.7 | 31.8 |
| Crevat-Pisano 1986 [[73](#_ENREF_73)] | 8 | 40 | 17-62 | 39.5 | 2094 | 67 | 25.4 |
| Patel 1990 [[74](#_ENREF_74)] | 6 | 40 | 42 (36-54) | 42 | 2224 | 73 | 33.1 |
| Bjorkman 1997 [[75](#_ENREF_75)] | 13 | 40 | 43 (28-55) | 43 | 2276 | 80.5 | 30.8 |
| Persson 1987 [[76](#_ENREF_76)] | 15 | 40 | 36-53 | 44.5 | 2354 | 66 | 28.3 |
| Malacrida 1991 [[77](#_ENREF_77)] | 8 | 40 | 50 (19-70) | 50 | 2640 | 66 | 24.9 |
| Bolon 2003 [[78](#_ENREF_78)] | 30 | 40 | 55 +/- 14 | 55 | 2900 | 68 | 31.3 |
| Barr 2001 [[79](#_ENREF_79)] | 9 | 40 | 63 +/- 10 | 63 | 3316 | 78 | 17.8 |
| Zomorodi 1998 [[80](#_ENREF_80)] | 63 | 40 | 63.7 | 63.7 | 3352.4 | 85.7 | 25.8 |
| Greenblatt 1984 [[67](#_ENREF_67)] | 9 | 40 | 67.6 +/- 1.4 | 67.6 | 3555.2 | 79.6 | 20.3 |
| Platten 1998 [[70](#_ENREF_70)] | 10 | 40 | 69 +/- 5 | 69 | 3628 | 70 | 20.6 |
| Greenblatt 1984 [[67](#_ENREF_67)] | 11 | 40 | 69.5 +/- 1.6 | 69.5 | 3654 | 59.3 | 25.9 |
| Albrecht 1999 [[64](#_ENREF_64)] | 9 | 40 | 71 (67-81) | 71 | 3732 | 80.5 | 23.3 |
| Smith 1984 [[81](#_ENREF_81)] | 13 | 40 | 71.3 (63-84) | 71.3 | 3747.6 | 61.5 | 18.5 |
| Bjorkman 2001 [[82](#_ENREF_82)] | 10 | 40 | 62-81 | 71.5 | 3758 | 68 | 35.5 |
| Kanto 1986 [[83](#_ENREF_83)] | 10 | 40 | 73.4 (67-79) | 73.4 | 3856.8 | 69.1 | 23.3 |

^a^ weight was estimated using the formula from [[39](#_ENREF_39)]; GA is gestational age, PNA is postnatal age, PMA is postmenstrual age, WT is weight, CL is clearance, range in (), +/- standard deviation.

**References**

1. Knight JA, Davis EM, Manouilov K, Hoie EB. The effect of postnatal age on gentamicin pharmacokinetics in neonates. Pharmacotherapy 2003; 23: 992-6.

2. Dodge WF, Jelliffe RW, Richardson CJ, McCleery RA, Hokanson JA, Snodgrass WR. Gentamicin population pharmacokinetic models for low birth weight infants using a new nonparametric method. Clin Pharmacol Ther 1991; 50: 25-31.

3. Nielsen EI, Sandstrom M, Honore PH, Ewald U, Friberg LE. Developmental pharmacokinetics of gentamicin in preterm and term neonates: population modelling of a prospective study. Clinical Pharmacokinetics 2009; 48: 253-63.

4. Low YS, Tan SL, Wan AS. Extended-interval gentamicin dosing in achieving therapeutic concentrations in malaysian neonates. J Pediatr Pharmacol Ther 2015; 20: 119-27.

5. Nakae S, Yamada M, Ito T, Chiba Y, Sasaki E, Sakamoto M, Tada K, Yamada T, Mori S. Gentamicin dosing and pharmacokinetics in low birth weight infants. Tohoku Journal of Experimental Medicine 1988; 155: 213-23.

6. Lulic-Botica M, Sheer T, Edwards D, Thomas RL, Natarajan G. Impact of small-for-gestational age (SGA) status on gentamicin pharmacokinetics in neonates. J Clin Pharmacol 2014; 54: 39-45.

7. DiCenzo R, Forrest A, Slish JC, Cole C, Guillet R. A gentamicin pharmacokinetic population model and once-daily dosing algorithm for neonates. Pharmacotherapy 2003; 23: 585-91.

8. Vervelde ML, Rademaker CM, Krediet TG, Fleer A, van Asten P, van Dijk A. Population pharmacokinetics of gentamicin in preterm neonates: evaluation of a once-daily dosage regimen. Therapeutic Drug Monitoring 1999; 21: 514-9.

9. Garcia B, Barcia E, Perez F, Molina IT. Population pharmacokinetics of gentamicin in premature newborns. Journal of Antimicrobial Chemotherapy 2006; 58: 372-9.

10. Lanao JM, Calvo MV, Mesa JA, Martin-Suarez A, Carbajosa MT, Miguelez F, Dominguez-Gil A. Pharmacokinetic basis for the use of extended interval dosage regimens of gentamicin in neonates. Journal of Antimicrobial Chemotherapy 2004; 54: 193-8.

11. Fuchs A, Guidi M, Giannoni E, Werner D, Buclin T, Widmer N, Csajka C. Population pharmacokinetic study of gentamicin in a large cohort of premature and term neonates. Br J Clin Pharmacol 2014; 78: 1090-101.

12. Haughey DB, Hilligoss DM, Grassi A, Schentag JJ. Two-compartment gentamicin pharmacokinetics in premature neonates: a comparison to adults with decreased glomerular filtration rates. Journal of Pediatrics 1980; 96: 325-30.

13. Botha JH, du Preez MJ, Adhikari M. Population pharmacokinetics of gentamicin in South African newborns. European Journal of Clinical Pharmacology 2003; 59: 755-9.

14. Lingvall M, Reith D, Broadbent R. The effect of sepsis upon gentamicin pharmacokinetics in neonates. British Journal of Clinical Pharmacology 2005; 59: 54-61.

15. Murphy JE, Austin ML, Frye RF. Evaluation of gentamicin pharmacokinetics and dosing protocols in 195 neonates. Am J Health Syst Pharm 1998; 55: 2280-8.

16. Thomson AH, Way S, Bryson SM, McGovern EM, Kelman AW, Whiting B. Population pharmacokinetics of gentamicin in neonates. Developmental Pharmacology and Therapeutics 1988; 11: 173-9.

17. Bhatt-Mehta V, Donn SM. Gentamicin pharmacokinetics in term newborn infants receiving high-frequency oscillatory ventilation or conventional mechanical ventilation: a case-controlled study. Journal of Perinatology 2003; 23: 559-62.

18. Izquierdo M, Lanao JM, Cervero L, Jimenez NV, Dominguez-Gil A. Population pharmacokinetics of gentamicin in premature infants. Therapeutic Drug Monitoring 1992; 14: 177-83.

19. Mark LF, Solomon A, Northington FJ, Lee CK. Gentamicin pharmacokinetics in neonates undergoing therapeutic hypothermia. Therapeutic Drug Monitoring 2013; 35: 217-22.

20. Lopez SA, Mulla H, Durward A, Tibby SM. Extended-interval gentamicin: population pharmacokinetics in pediatric critical illness. Pediatric Critical Care Medicine 2010; 11: 267-74.

21. Cohen P, Collart L, Prober CG, Fischer AF, Blaschke TF. Gentamicin pharmacokinetics in neonates undergoing extracorporal membrane oxygenation. Pediatric Infectious Disease Journal 1990; 9: 562-6.

22. Medellin-Garibay SE, Rueda-Naharro A, Pena-Cabia S, Garcia B, Romano-Moreno S, Barcia E. Population pharmacokinetics of gentamicin and dosing optimization for infants. Antimicrobial Agents and Chemotherapy 2015; 59: 482-9.

23. Bass KD, Larkin SE, Paap C, Haase GM. Pharmacokinetics of once-daily gentamicin dosing in pediatric patients. Journal of Pediatric Surgery 1998; 33: 1104-7.

24. Shankar SM, Jew RK, Bickert BM, Cavalieri GE, Bell LM, Lange BJ. Pharmacokinetics of single daily dose gentamicin in children with cancer. J Pediatr Hematol Oncol 1999; 21: 284-8.

25. Ho KK, Bryson SM, Thiessen JJ, Greenberg ML, Einarson TR, Leson CL. The effects of age and chemotherapy on gentamicin pharmacokinetics and dosing in pediatric oncology patients. Pharmacotherapy 1995; 15: 754-64.

26. Postovsky S, Ben Arush MW, Kassis E, Elhasid R, Krivoy N. Pharmacokinetic analysis of gentamicin thrice and single daily dosage in pediatric cancer patients. Pediatric Hematology and Oncology 1997; 14: 547-54.

27. Walker JM, Wise R, Mitchard M. The pharmacokinetics of amikacin and gentamicin in volunteers: a comparison of individual differences. Journal of Antimicrobial Chemotherapy 1979; 5: 95-9.

28. el-Sayed YM, Islam SI. Effect of age and renal function on gentamicin pharmacokinetic parameters. Int J Clin Pharmacol Ther Toxicol 1989; 27: 503-9.

29. Zaske DE, Cipolle RJ, Rotschafer JC, Solem LD, Mosier NR, Strate RG. Gentamicin pharmacokinetics in 1,640 patients: method for control of serum concentrations. Antimicrobial Agents and Chemotherapy 1982; 21: 407-11.

30. Simon VK, Mosinger EU, Malerczy V. Pharmacokinetic studies of tobramycin and gentamicin. Antimicrobial Agents and Chemotherapy 1973; 3: 445-50.

31. Demczar DJ, Nafziger AN, Bertino JS, Jr. Pharmacokinetics of gentamicin at traditional versus high doses: implications for once-daily aminoglycoside dosing. Antimicrobial Agents and Chemotherapy 1997; 41: 1115-9.

32. Gilman TM, Brunnemann SR, Segal JL. Comparison of population pharmacokinetic models for gentamicin in spinal cord-injured and able-bodied patients. Antimicrobial Agents and Chemotherapy 1993; 37: 93-9.

33. Bianco TM, Dwyer PN, Bertino JS, Jr. Gentamicin pharmacokinetics, nephrotoxicity, and prediction of mortality in febrile neutropenic patients. Antimicrobial Agents and Chemotherapy 1989; 33: 1890-5.

34. Bertino JS, Jr., Booker LA, Franck P, Rybicki B. Gentamicin pharmacokinetics in patients with malignancies. Antimicrobial Agents and Chemotherapy 1991; 35: 1501-3.

35. Lewis DR, Longman RJ, Wisheart JD, Spencer RC, Brown NM. The pharmacokinetics of a single dose of gentamicin (4 mg/kg) as prophylaxis in cardiac surgery requiring cardiopulmonary bypass. Cardiovascular Surgery 1999; 7: 398-401.

36. Goncalves-Pereira J, Martins A, Povoa P. Pharmacokinetics of gentamicin in critically ill patients: pilot study evaluating the first dose. Clinical Microbiology and Infection 2010; 16: 1258-63.

37. Hilmer SN, Tran K, Rubie P, Wright J, Gnjidic D, Mitchell SJ, Matthews S, Carroll PR. Gentamicin pharmacokinetics in old age and frailty. British Journal of Clinical Pharmacology 2011; 71: 224-31.

38. Johnston C, Hilmer SN, McLachlan AJ, Matthews ST, Carroll PR, Kirkpatrick CM. The impact of frailty on pharmacokinetics in older people: using gentamicin population pharmacokinetic modeling to investigate changes in renal drug clearance by glomerular filtration. European Journal of Clinical Pharmacology 2014; 70: 549-55.

39. Sumpter AL, Holford NH. Predicting weight using postmenstrual age--neonates to adults. Paediatric Anaesthesia 2011; 21: 309-15.

40. Lee TC, Charles BG, Harte GJ, Gray PH, Steer PA, Flenady VJ. Population pharmacokinetic modeling in very premature infants receiving midazolam during mechanical ventilation: midazolam neonatal pharmacokinetics. Anesthesiology 1999; 90: 451-7.

41. Harte GJ, Gray PH, Lee TC, Steer PA, Charles BG. Haemodynamic responses and population pharmacokinetics of midazolam following administration to ventilated, preterm neonates. Journal of Paediatrics and Child Health 1997; 33: 335-8.

42. de Wildt SN, Kearns GL, Hop WC, Murry DJ, Abdel-Rahman SM, van den Anker JN. Pharmacokinetics and metabolism of intravenous midazolam in preterm infants. Clinical Pharmacology and Therapeutics 2001; 70: 525-31.

43. Jacqz-Aigrain E, Daoud P, Burtin P, Maherzi S, Beaufils F. Pharmacokinetics of midazolam during continuous infusion in critically ill neonates. European Journal of Clinical Pharmacology 1992; 42: 329-32.

44. Burtin P, Jacqz-Aigrain E, Girard P, Lenclen R, Magny JF, Betremieux P, Tehiry C, Desplanques L, Mussat P. Population pharmacokinetics of midazolam in neonates. Clinical Pharmacology and Therapeutics 1994; 56: 615-25.

45. Jacqz-Aigrain E, Wood C, Robieux I. Pharmacokinetics of midazolam in critically ill neonates. European Journal of Clinical Pharmacology 1990; 39: 191-2.

46. Mulla H, McCormack P, Lawson G, Firmin RK, Upton DR. Pharmacokinetics of midazolam in neonates undergoing extracorporeal membrane oxygenation. Anesthesiology 2003; 99: 275-82.

47. Ahsman MJ, Hanekamp M, Wildschut ED, Tibboel D, Mathot RA. Population pharmacokinetics of midazolam and its metabolites during venoarterial extracorporeal membrane oxygenation in neonates. Clinical Pharmacokinetics 2010; 49: 407-19.

48. Peeters MY, Prins SA, Knibbe CA, Dejongh J, Mathot RA, Warris C, van Schaik RH, Tibboel D, Danhof M. Pharmacokinetics and pharmacodynamics of midazolam and metabolites in nonventilated infants after craniofacial surgery. Anesthesiology 2006; 105: 1135-46.

49. Reed MD, Rodarte A, Blumer JL, Khoo KC, Akbari B, Pou S, Pharmd, Kearns GL. The single-dose pharmacokinetics of midazolam and its primary metabolite in pediatric patients after oral and intravenous administration. Journal of Clinical Pharmacology 2001; 41: 1359-69.

50. Mathews HM, Carson IW, Lyons SM, Orr IA, Collier PS, Howard PJ, Dundee JW. A pharmacokinetic study of midazolam in paediatric patients undergoing cardiac surgery. British Journal of Anaesthesia 1988; 61: 302-7.

51. Muchohi SN, Kokwaro GO, Ogutu BR, Edwards G, Ward SA, Newton CR. Pharmacokinetics and clinical efficacy of midazolam in children with severe malaria and convulsions. British Journal of Clinical Pharmacology 2008; 66: 529-38.

52. Rey E, Delaunay L, Pons G, Murat I, Richard MO, Saint-Maurice C, Olive G. Pharmacokinetics of midazolam in children: comparative study of intranasal and intravenous administration. European Journal of Clinical Pharmacology 1991; 41: 355-7.

53. Payne K, Mattheyse FJ, Liebenberg D, Dawes T. The pharmacokinetics of midazolam in paediatric patients. European Journal of Clinical Pharmacology 1989; 37: 267-72.

54. Salonen M, Kanto J, Iisalo E, Himberg JJ. Midazolam as an induction agent in children: a pharmacokinetic and clinical study. Anesthesia and Analgesia 1987; 66: 625-8.

55. Tolia V, Brennan S, Aravind MK, Kauffman RE. Pharmacokinetic and pharmacodynamic study of midazolam in children during esophagogastroduodenoscopy. Journal of Pediatrics 1991; 119: 467-71.

56. Mandema JW, Tuk B, van Steveninck AL, Breimer DD, Cohen AF, Danhof M. Pharmacokinetic-pharmacodynamic modeling of the central nervous system effects of midazolam and its main metabolite alpha-hydroxymidazolam in healthy volunteers. Clin Pharmacol Ther 1992; 51: 715-28.

57. Olkkola KT, Ahonen J, Neuvonen PJ. The effects of the systemic antimycotics, itraconazole and fluconazole, on the pharmacokinetics and pharmacodynamics of intravenous and oral midazolam. Anesth Analg 1996; 82: 511-6.

58. Wermeling DP, Record KA, Kelly TH, Archer SM, Clinch T, Rudy AC. Pharmacokinetics and pharmacodynamics of a new intranasal midazolam formulation in healthy volunteers. Anesth Analg 2006; 103: 344-9, table of contents.

59. Shord SS, Chan LN, Camp JR, Vasquez EM, Jeong HY, Molokie RE, Baum CL, Xie H. Effects of oral clotrimazole troches on the pharmacokinetics of oral and intravenous midazolam. Br J Clin Pharmacol 2010; 69: 160-6.

60. Breimer LT, Hennis PJ, Burm AG, Danhof M, Bovill JG, Spierdijk J, Vletter AA. Quantification of the EEG effect of midazolam by aperiodic analysis in volunteers. Pharmacokinetic/pharmacodynamic modelling. Clin Pharmacokinet 1990; 18: 245-53.

61. Heizmann P, Eckert M, Ziegler WH. Pharmacokinetics and bioavailability of midazolam in man. British Journal of Clinical Pharmacology 1983; 16 Suppl 1: 43S-9S.

62. Wong SL, Goldberg MR, Ballow CH, Kitt MM, Barriere SL. Effect of Telavancin on the pharmacokinetics of the cytochrome P450 3A probe substrate midazolam: a randomized, double-blind, crossover study in healthy subjects. Pharmacotherapy 2010; 30: 136-43.

63. Schwagmeier R, Alincic S, Striebel HW. Midazolam pharmacokinetics following intravenous and buccal administration. British Journal of Clinical Pharmacology 1998; 46: 203-6.

64. Albrecht S, Ihmsen H, Hering W, Geisslinger G, Dingemanse J, Schwilden H, Schuttler J. The effect of age on the pharmacokinetics and pharmacodynamics of midazolam. Clinical Pharmacology and Therapeutics 1999; 65: 630-9.

65. Clausen TG, Wolff J, Hansen PB, Larsen F, Rasmussen SN, Dixon JS, Crevoisier C. Pharmacokinetics of midazolam and alpha-hydroxy-midazolam following rectal and intravenous administration. British Journal of Clinical Pharmacology 1988; 25: 457-63.

66. van Gerven JM, Roncari G, Schoemaker RC, Massarella J, Keesmaat P, Kooyman H, Heizmann P, Zell M, Cohen AF, Dingemanse J. Integrated pharmacokinetics and pharmacodynamics of Ro 48-8684, a new benzodiazepine, in comparison with midazolam during first administration to healthy male subjects. British Journal of Clinical Pharmacology 1997; 44: 487-93.

67. Greenblatt DJ, Abernethy DR, Locniskar A, Harmatz JS, Limjuco RA, Shader RI. Effect of age, gender, and obesity on midazolam kinetics. Anesthesiology 1984; 61: 27-35.

68. Balson KR, Morgan DJ, Richmond BH, McAlindon ME, Elliott SL, Yeomans ND. Pharmacokinetics of midazolam in Vietnamese subjects. J Gastroenterol Hepatol 1996; 11: 1177-80.

69. Hostler D, Zhou J, Tortorici MA, Bies RR, Rittenberger JC, Empey PE, Kochanek PM, Callaway CW, Poloyac SM. Mild hypothermia alters midazolam pharmacokinetics in normal healthy volunteers. Drug Metab Dispos 2010; 38: 781-8.

70. Platten HP, Schweizer E, Dilger K, Mikus G, Klotz U. Pharmacokinetics and the pharmacodynamic action of midazolam in young and elderly patients undergoing tooth extraction. Clin Pharmacol Ther 1998; 63: 552-60.

71. Bancke LL, Dworak HA, Rodvold KA, Halvorsen MB, Gidal BE. Pharmacokinetics, pharmacodynamics, and safety of USL261, a midazolam formulation optimized for intranasal delivery, in a randomized study with healthy volunteers. Epilepsia 2015; 56: 1723-31.

72. Garg V, Chandorkar G, Farmer HF, Smith F, Alves K, van Heeswijk RP. Effect of telaprevir on the pharmacokinetics of midazolam and digoxin. J Clin Pharmacol 2012; 52: 1566-73.

73. Crevat-Pisano P, Dragna S, Granthil C, Coassolo P, Cano JP, Francois G. Plasma concentrations and pharmacokinetics of midazolam during anaesthesia. J Pharm Pharmacol 1986; 38: 578-82.

74. Patel IH, Soni PP, Fukuda EK, Smith DF, Leier CV, Boudoulas H. The pharmacokinetics of midazolam in patients with congestive heart failure. British Journal of Clinical Pharmacology 1990; 29: 565-9.

75. Bjorkman S, Rigemar G, Idvall J. Pharmacokinetics of midazolam given as an intranasal spray to adult surgical patients. British Journal of Anaesthesia 1997; 79: 575-80.

76. Persson P, Nilsson A, Hartvig P, Tamsen A. Pharmacokinetics of midazolam in total i.v. anaesthesia. British Journal of Anaesthesia 1987; 59: 548-56.

77. Malacrida R, Fritz ME, Suter PM, Crevoisier C. Pharmacokinetics of midazolam administered by continuous intravenous infusion to intensive care patients. Critical Care Medicine 1992; 20: 1123-6.

78. Bolon M, Bastien O, Flamens C, Paulus S, Salord F, Boulieu R. Evaluation of the estimation of midazolam concentrations and pharmacokinetic parameters in intensive care patients using a bayesian pharmacokinetic software (PKS) according to sparse sampling approach. J Pharm Pharmacol 2003; 55: 765-71.

79. Barr J, Zomorodi K, Bertaccini EJ, Shafer SL, Geller E. A double-blind, randomized comparison of i.v. lorazepam versus midazolam for sedation of ICU patients via a pharmacologic model. Anesthesiology 2001; 95: 286-98.

80. Zomorodi K, Donner A, Somma J, Barr J, Sladen R, Ramsay J, Geller E, Shafer SL. Population pharmacokinetics of midazolam administered by target controlled infusion for sedation following coronary artery bypass grafting. Anesthesiology 1998; 89: 1418-29.

81. Smith MT, Heazlewood V, Eadie MJ, Brophy TO, Tyrer JH. Pharmacokinetics of midazolam in the aged. Eur J Clin Pharmacol 1984; 26: 381-8.

82. Bjorkman S, Wada DR, Berling BM, Benoni G. Prediction of the disposition of midazolam in surgical patients by a physiologically based pharmacokinetic model. Journal of Pharmaceutical Sciences 2001; 90: 1226-41.

83. Kanto J, Aaltonen L, Himberg JJ, Hovi-Viander M. Midazolam as an intravenous induction agent in the elderly: a clinical and pharmacokinetic study. Anesthesia and Analgesia 1986; 65: 15-20.
